# HIV-1 genotype diversity and distribution characteristics among heterosexually transmitted population in Jiangsu province, China

**DOI:** 10.1186/s12985-019-1162-4

**Published:** 2019-04-25

**Authors:** Peipei Xiao, Ying Zhou, Jing Lu, Li Yan, Xiaoqin Xu, Haiyang Hu, Jianjun Li, Ping Ding, Tao Qiu, Gengfeng Fu, Xiping Huan, Haitao Yang

**Affiliations:** 1 Department of Epidemiology and Health Statistics, School of Public Health, Southeast University|, No.87 Dingjiaqiao, Nanjing, 210009 China; 2 Department of HIV/STD Prevention and Control, Jiangsu Provincial Center for Disease Prevention and Control, No.172 Jiangsu Road, Nanjing, 210009 China; 3 Jiangsu Research Institute of Schistosomiasis Control, No.117 Meiyuan Yangxiang, Wuxi, 214064 China

**Keywords:** HIV-1, Genotype, Heterosexual transmission, Distribution, China

## Abstract

**Background:**

Heterosexual transmission has contributed greatly to the current HIV-1 epidemic in China. However, the HIV-1 genetic characteristics in the heterosexually transmitted population in Jiangsu province remained unclear.

**Methods:**

A molecular epidemiological investigation on heterosexual transmission of HIV-1 was conducted across Jiangsu province. 301 HIV-1 patients infected through heterosexual transmission were involved in this study. The epidemiological information was investigated by trained staff via face-to-face interviews. Blood samples were taken from each patient, HIV-1 RNA was extracted from the plasma, and used for amplifying the *gag* and *env* genes followed by further products sequencing. The genotypes of HIV-1 were determined using phylogenetic tree analyses in the neighbor-joining method.

**Results:**

A total of 262 samples were successfully taken for genotyping. The main subtypes which accounted for 90.5% of all HIV-1 strains are CRF01_AE (45.4%), CRF07_BC (21.4%), subtype B (12.6%), CRF08_BC (11.1%). Minor subtypes were also detected, such as CRF68_01B, subtype C, CRF55_01B, CRF02_AG and subtype A. Time trend analysis suggested the prevalence of subtype B and CRF08_BC decreased gradually, but the prevalence of CRF01_AE increased over time. A relatively higher prevalence of CRF07_BC in Central Jiangsu and subtype B were detected in South Jiangsu, while a relatively lower prevalence of subtype B and CRF08_BC were detected in Central Jiangsu.

**Conclusion:**

Complex and unbalanced HIV distribution characteristics suggest that heterosexual transmission of HIV needs to be taken seriously. It is necessary to implement more effective and comprehensive intervention strategies for further control of HIV-1 dissemination.

## Background

The HIV/AIDS epidemic in China continues to pose a major public health threat. By the end of September, 2018, It was reported that 849,602 individuals have contracted HIV and 262,442 have died from AIDS-related illnesses in China [1]. HIV has historically been restrained to certain high-risk groups, such as intravenous drug users (IDUs) and former plasma donors (FPDs) [2]. With a gradual shift in the driving force of China’s HIV epidemic, sexual transmission has become the predominant route of HIV infection currently in China [3]. Heterosexual transmission cases accounted for 71.1% of all newly diagnosed HIV infections by the end of September, 2018 [1]. HIV-1 exhibits an extensive genetic diversity due to its high capacity for mutation, recombination and replication. Due to the resulting high genetic variation, HIV-1 can be classified into many different groups or subtypes including circulating recombinant forms (CRFs) and unique recombinant forms (URFs) [4]. A recent study showed that 8 subtypes and 21 CRFs have been identified in China since 2013. CRF01_AE, CRF07_BC, subtype B, CRF08_BC, subtype C were the four main subtypes, accounting for 95.1% of all HIV-1 infections [5]. HIV subtypes have been changing over the past three decades in different risk groups and regions in China. Consequently, conducting HIV molecular epidemiological surveillance is important for understanding the epidemic of HIV.

Jiangsu province is located in the southeast coastal area of China with a residential population of 80 million. As one of the three most economically developed regions in China, it attracts a huge number of immigrants to work and settle in the region [6]. By September 2018, a total of 24,592 HIV-positive cases have been reported in Jiangsu. Among the newly diagnosed HIV-1 cases in 2018, 97.9% were infected through sexual transmission, of which homosexual and heterosexual transmission accounted for 54.2 and 43.2%, respectively [7]. A previous study showed that a majority of HIV-1 strains circulating in Jiangsu were CRF01_AE, subtype B and CRF07_BC [8]. Growing information in recent years strongly suggested that unprotected sex behavior among high-risk groups were the “incubator” and “accelerator” of HIV-1 genetic diversity in China [9], and HIV-1 genotypes possibly change accordingly with its shift in transmission modes. Currently, many researchers concentrate on men who have sex with men (MSM) population due to rapid increasing HIV incidence. However, very few studies have been conducted to illustrate HIV-1 subtypes circulating in heterosexuals, which remains the primary mode of transmission. In recent years, there are signs that heterosexual transmission is posing a risk bridge spreading HIV-1 from specific high-risk groups into the general public, which might increase the complexity and diversity of HIV-1 genotypes [5, 10, 11]. Concurrently, frequent population migration may accelerate the spreading speed and co-circulation of various HIV-1 subtypes in this area. Some novel HIV-1 recombinants have been previously reported in heterosexuals in Jiangsu and its surrounding provinces [12, 13]. Therefore, it is necessary to perform a detailed analysis of HIV-1 subtypes and its distribution characteristics among heterosexuals.

Currently, the HIV-1 genetic characteristics of HIV in the population infected through heterosexual transmission are still insufficient in the Chinese database and even unavailable with Jiangsu profiles. In the present study, we focused on HIV-1 infection through heterosexual transmission and conducted a molecular epidemiological investigation to reveal the HIV-1 genotypes and its distribution characteristics in Jiangsu province.

## Methods

### The study subjects and specimens

The selection criteria for subjects and samples of this study are as follows: (1) Cases of HIV infections were collected with follow-up and regular testing for CD4+ T cells count and viral load from May to August, 2017 at the Center of Jiangsu Provincial HIV/AIDS confirmatory laboratory, Jiangsu Provincial Center for Disease Control and Prevention (CDC); (2) The collected samples which tested for viral load > 1000 copies/mL were selected for enrolment; (3) The subjects must be > 18 years old; (4) The subject was infected with HIV via heterosexual contact as determined by interview; (5) The subjects have given informed consent, volunteered and were able to complete the questionnaire. To reduce reporting bias, cases with uncertain HIV-infected pathways or a history of mixed high-risk behaviors (such as bisexual behavior or sexual behavior with injecting drug use) were excluded from the study. Each participant completed the questionnaire with a trained and experienced investigator in a single room. The epidemiological information was investigated via face-to-face interviews, using a standardized CDC-administered questionnaire (eg. describing gender, age, occupation, education, marital status, level of income, geographic location, high-risk behaviors, mode of transmission) by trained healthcare workers in the local CDC. 5 mL of whole blood was drawn from each participant using an EDTA-K3 vacuum tube (BD Company, USA). All plasma was separated by centrifugation at 3000 rpm for 15 min from the whole blood samples and stored in a − 80 °C freezer until being used for subsequent analysis (HIV-1 RNA *gag* and *env* gene sequences) in Jiangsu CDC.

### Amplification and sequencing of HIV-1 *gag* and *env* gene fragments

HIV-1 RNA was extracted from the patients’ plasma (140 μL) using the QIAamp Viral RNA Mini kit (Qiagen, Gmbh, Hilden, Germany) according to the manufacturer’s instructions and then subjected to the amplification of HIV-1 *gag* gene (HXB2: 781–1861) and *env* gene (HXB2: 6540–7384) for HIV-1 genotyping. The *gag* gene fragment was amplified in a one-step reverse transcription polymerase chain reaction (RT-PCR) with primers GAG-L (5′TCGACGCAGGACTCGGCTTGC-3′, sense) and GAG-E2 (5′-TCCAACAGCCCTTTTTCCTAGG-3′, antisense) in a 25 μL reaction volume. The first round of reaction conditions were as follows: one cycle of 50 °C for 30 min, 94 °C for 5 min, 55 °C for 1 min and 72 °C for 2 min; then 35 cycles of 94 °C for 30 s, 55 °C for 45 s and 72 °C for 1.5 min; and followed by 72 °C for 10 min, and holding at 4 °C. The second round of *gag* PCR (Nested Polymerase Chain Reaction, Nested-PCR) was performed using 2 × Taq PCR MasterMix (Takara) and primers GUX (5′-AGGAGAGAGATGGGTGCGAGAGCGTC-3′, sense) and GDX (5′-GGCTAGTTCCTCCTACTCCCTGACAT-3′, antisense) in a 50 μL reaction volume. Cycling conditions were: one cycle of 94 °C for 2 min, 55 °C for 1 min and 72 °C for 1.5 min; followed by 35 cycles of 94 °C for 30 s, 55 °C for 45 s and 72 °C for 1.5 min; and finally, 72 °C for 10 min, and holding at 4 °C. The *env* gene fragment was amplified by RT-PCR using primers enF1 (5′-GATGCATGAGGATATAATCAGTTTATGGGA-3′, sense) and enR1 (5′-ATTGATGCTGCGCCCATAGTGCT-3′, antisense) in a 25 μL reaction volume, and nested PCR was implemented with primers EC1FA (5′-GAGGATRTAATCAGTTTATGGGATC-3′, sense) and EC3RA (5′-GTATTRCAATAGAAAAATTCYCCTC-3′, antisense) in a 50 μL reaction volume, respectively. The reaction cycling conditions were the same as mentioned above. Negative controls were established at each step to avoid potential laboratory contamination. PCR products were identified by 1% agarose gel electrophoresis. Finally, the amplified positive products were sent to SinoGenoMax Biotechnology Company (Beijing, China) for sequencing.

### HIV-1 genotyping and phylogenetic analyzes

The obtained sequence fragments in *gag* and *env* gene regions were edited and assembled using ChromasPro 1.6. All assembled sequences were aligned together with the reference sequences using Clustal W program implemented in MAGE 7.0 (available at: http://www.megasoftware.net/) software [14], and then further checked manually in Bioedit. Phylogenetic trees were constructed using the neighbor-joining method based on the Kimura two-parameter model with MEGA 7.0 [15]. The significance of branch orders was tested by bootstrapping analysis with 1000 replicates. Additionally, to check for potential errors, the sequences obtained were also subjected to the online HIV Basic Local Alignment Search Tool (BLAST) prior to analysis to compare with all known sequences in the HIV Sequence Database from Los Alamos National Laboratory (https://www.hiv.lanl.gov/content/sequence/BASIC_BLAST/basic_blast.html). The HIV-1 subtypes of each patient was determined based on the genotypes of both the *gag* and *env* gene regions. If only one gene region was available, the subtype of that region was designated. Samples with different genotypic identification from the *gag* and *env* regions were provisionally considered as URFs, and labelled by the *gag*/*env* subtype designations. For example, CRF01_AE/CRF07_BC indicated that CRF01_AE in *gag* gene and CRF07_BC in *env* gene region were identified.

### Statistical analysis

The categorical variables were calculated as absolute values and percentages, Pearson’s Chi squared test or Fisher exact test was used for analysis when more than 20% of cells had an expected count ≤5. Statistical analyses were implemented using SPSS 20.0 (IBM Inc., New York, NY, USA). All probability values were two-tailed, and variables with *P* < 0.05 was considered to have statistical significance.

## Results

### Sample amplification status and characteristics of enrolled subjects

**Fig. 1:**
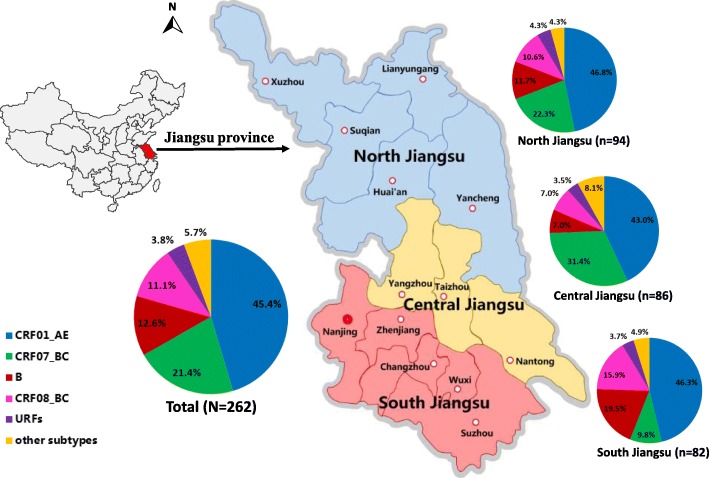
The geographic location of 13 prefectures and HIV-1 genotyping distribution among heterosexuals in Jiangsu province, China. South Jiangsu includes Suzhou, Wuxi, Changzhou, Zhenjiang and Nanjing cities; Central Jiangsu includes Nantong, Taizhou and Yangzhou cities; North Jiangsu includes Xuzhou, Suqian, Huai’an, Lianyungang and Yancheng cities. Other subtypes include subtype C, CRF02_AG, CRF55_01B, CRF68_01B and subtype A

Three hundred one subjects and their whole blood samples were included in this study, all subjects were from 13 prefectures of Jiangsu, the geographic location of 13 prefectures in Jiangsu province was shown in Fig. 1. Of the 301 samples included in this study, 262 samples (87.0%, 262/301) were obtained and successfully genotyped based on the overall analyses of *gag* and *env* gene regions. Sociodemographic characteristics of the participants are showed in Table 1. The majority of participants are male (71.8%) and registered their household locally in Jiangsu (85.5%). More than half of them (61.8%) were 31 to 49 years old. 49.2% of subjects work full-time and 65.6% had a monthly income of less than 3000 CNY. 59.2% of subjects have been married, and 88.5% had an education of middle school or below. Among the three sources of transmission, commercial sexual contact (44.7%) was the major risk factor for HIV-1 infection.

**Table 1:** The distribution characteristics of HIV-1 genotypes among the heterosexually transmitted population in Jiangsu

Characteristics	Cases[%]^a^	The frequency and prevalence of different HIV-1 genotypes, *N*(%)^b^						*P*
		CRF01_AE	CRF07_BC	B	CRF08_BC	URFs	Other subtypes^d^	
Total	262 [100]	119 (45.4)	56 (21.4)	33 (12.6)	29 (11.1)	10 (3.8)	15 (5.7)	
Gender								0.001
Male	188 [71.8]	88 (46.8)	41 (21.8)	23 (12.2)	12 (6.4)	10 (5.3)	14 (7.4)	
Female	74 [28.2]	31 (41.9)	15 (20.3)	10 (13.5)	17 (23.0)	0 (0.0)	1 (1.4)	
Transmission routes								0.074^**c**^
Marital transmission	63 [24.0]	28 (44.4)	11 (17.5)	10 (15.9)	11 (17.5)	1 (1.6)	2 (3.2)	
Extra-marital non-commercial sexual contact	82 [31.3]	33 (40.2)	16 (19.5)	15 (18.3)	11 (13.4)	2 (2.4)	5 (6.1)	
Commercial sexual contact	117 [44.7]	58 (49.6)	29 (24.8)	8 (6.8)	7 (6.0)	7 (6.0)	8 (6.8)	
HIV-1 confirmation year								0.028^**c**^
Before 2008	24 [9.2]	4 (16.7)	5 (20.8)	8 (33.3)	4 (16.7)	0 (0.0)	3 (12.5)	
2008–2012	138 [52.7]	63 (45.7)	28 (20.3)	18 (13.0)	16 (11.6)	6 (4.3)	7 (5.1)	
After 2012	100 [38.2]	52 (52.0)	23 (23.0)	7 (7.0)	9 (9.0)	4 (4.0)	5 (5.0)	
Ages (years)								0.183^**c**^
18–30	32 [12.2]	15 (46.9)	7 (21.9)	4 (12.5)	3 (9.4)	2 (6.2)	1 (3.1)	
31–49	162 [61.8]	75 (46.3)	30 (18.5)	17 (10.5)	19 (11.7)	7 (4.3)	14 (8.6)	
≥ 50	68 [26.0]	29 (42.6)	19 (27.9)	12 (17.6)	7 (10.3)	1 (1.5)	0 (0.0)	
Marital status								0.453
Married	155 [59.2]	65 (41.9)	34 (21.9)	17 (11.0)	23 (14.8)	6 (3.9)	10 (6.5)	
Unmarried	32 [12.2]	19 (59.4)	7 (21.9)	4 (12.5)	1 (3.1)	1 (3.1)	0 (0.0)	
Divorced/widowed	75 [28.6]	35 (46.7)	15 (20.0)	12 (16.0)	5 (6.7)	3 (4.0)	5 (6.7)	
Education level								0.533
Primary school and below	59 [22.5]	29 (49.2)	13 (22.0)	10 (16.9)	5 (8.5)	1 (1.7)	1 (1.7)	
Middle school	173 [66.0]	76 (43.9)	38 (22.0)	21 (12.1)	21 (12.1)	6 (3.5)	11 (6.4)	
College and above	30 [11.5]	14 (46.7)	5 (16.7)	2 (6.7)	3 (10.0)	3 (10.0)	3 (10.0)	
Occupation								0.005^**c**^
Full-time job	129 [49.2]	64 (49.6)	23 (17.8)	10 (7.8)	16 (12.4)	6 (4.7)	10 (7.8)	
Part-time job	37 [14.1]	17 (45.9)	9 (24.3)	3 (8.1)	2 (5.4)	3 (8.1)	3 (8.1)	
Farmer	31 [11.8]	8 (25.8)	6 (19.4)	9 (29.0)	8 (25.8)	0 (0.0)	0 (0.0)	
Unemployed/retired	65 [24.8]	30 (46.2)	18 (27.7)	11 (16.9)	3 (4.6)	1 (1.5)	2 (3.1)	
Local census register								0.201
No	38 [14.5]	17 (44.7)	4 (10.5)	5 (13.2)	8 (21.1)	1 (2.6)	3 (7.9)	
Yes	224 [85.5]	102 (45.5)	52 (23.2)	28 (12.5)	21 (9.4)	9 (4.0)	12 (5.4)	
Monthly income level (CNY)								0.345
< 3000	172 [65.6]	79 (45.9)	38 (22.1)	24 (14.0)	20 (11.6)	4 (2.3)	7 (4.1)	
3000–5000	65 [24.8]	25 (38.5)	14 (21.5)	8 (12.3)	7 (10.8)	4 (6.2)	7 (10.8)	
> 5000	25 [9.5]	15 (60.0)	4 (16.0)	1 (4.0)	2 (8.0)	2 (8.0)	1 (4.0)	
Locations								0.035^**c**^
South Jiangsu	82 [31.3]	38 (46.3)	8 (9.8)	16 (19.5)	13 (15.9)	3 (3.7)	4 (4.9)	
Central Jiangsu	86 [32.8]	37 (43.0)	27 (31.4)	6 (7.0)	6 (7.0)	3 (3.5)	7 (8.1)	
North Jiangsu	94 [35.9]	44 (46.8)	21 (22.3)	11 (11.7)	10 (10.6)	4 (4.3)	4 (4.3)	

^a^Numbers in square brackets indicate the proportion of the HIV-1 cases as a percentage of the total 262 subjects

^b^Numbers in parentheses indicate the proportion of HIV-1 subtypes as a percentage of each subgroup

^c^The *P* value was calculated using Fisher’s exact method

^d^Other subtypes including CRF68_01B (2.7%), subtype C (1.9%), CRF02_AG (0.4%), CRF55_01B (0.4%) and subtype A (0.4%)

### HIV-1 genotyping analysis

**Fig. 2:**
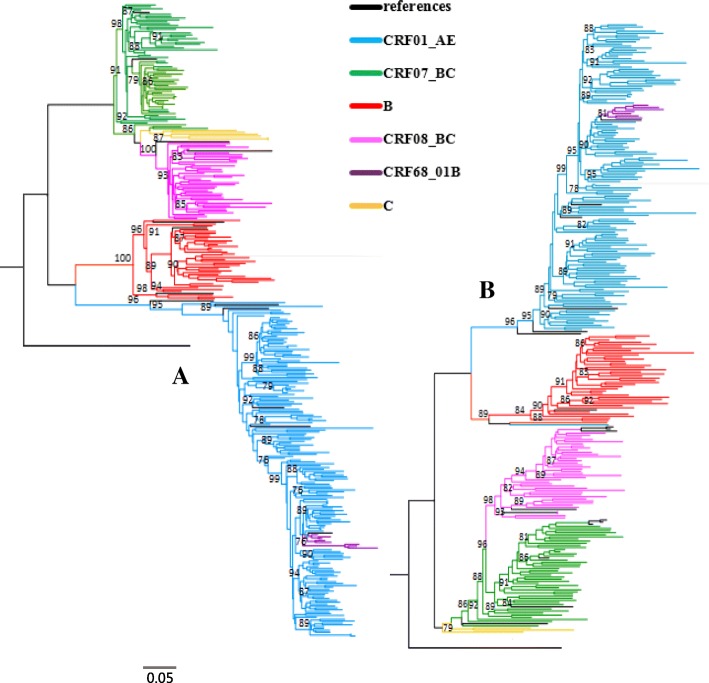
Phylogenetic tree analysis of *gag* gene (**a**) and *env* gene (**b**) region sequences from heterosexual transmitted HIV-1 individuals in Jiangsu

As showed in Table 1, CRF01_AE was found dominant (45.4%, 119/262), followed by CRF07_BC (21.4%, 56/262), subtype B (12.6%, 33/262), CRF08_BC (11.1%, 29/262), URFs (3.8%, 10/262), CRF68_01B (2.7%, 7/262), subtype C (1.9%, 5/262), CRF02_AG (0.4%, 1/262), CRF55_01B (0.4%, 1/262), and subtype A (0.4%, 1/262). As shown in Fig. 2, the phylogenetic tree of HIV-1 *gag* and *env* gene sequences was constructed using the neighbor-joining method. Additionally, 10 URFs samples with different genotypic identification from the *gag* and *env* regions were observed, with detailed information for these samples shown in Table 2.

**Table 2:** Several potential URFs information from different genotypic identification in *gag* and *env* regions

Sample ID	Sex	Transmission route	Age	Marital status	Number of heterosexual partner	region	*gag* gene genotype	*env* gene genotype
JS02G007	male	commercial sex contact	43	married	2–5	Lianyungang	CRF01_AE	CRF07_BC
JS02G008	male	commercial sex contact	28	married	2–5	Lianyungang	CRF01_AE	CRF07_BC
JS02L028	male	commercial sex contact	33	divorced	2–5	Zhenjiang	CRF07_BC	CRF01_AE
JS02L033	male	marital transmission	25	married	1	Zhenjiang	CRF08_BC	CRF07_BC
JS01C011	male	commercial sex contact	48	married	2–5	Xuzhou	B	CRF01_AE
JS02C002	male	commercial sex contact	42	widowed	6–10	Xuzhou	CRF08_BC	CRF07_BC
JS02M027	male	extra-marital non-commercial sexual contact	31	unmarried	2–5	Taizhou	BC	CRF01_AE
JS02B001	male	commercial sex contact	62	married	2–5	Wuxi	CRF01_AE	B
JS02F025	male	commercial sex contact	35	divorced	2–5	Nantong	CRF07_BC	CRF01_AE
JS02F055	male	commercial sex contact	40	married	2–5	Nantong	CRF01_AE	CRF07_BC

### The distribution characteristics of HIV-1 genotypes

**Fig. 3:**
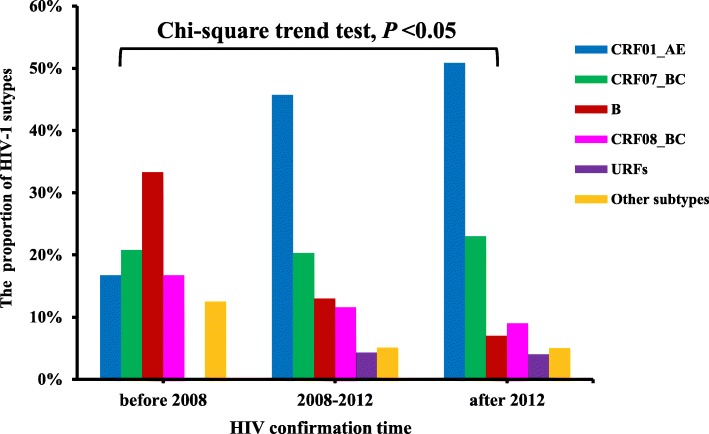
The time distribution of HIV-1 subtypes among heterosexuals in Jiangsu

As revealed in Table 1, The HIV-1 genotypes distribution showed significant statistical differences by gender, HIV confirmation year, occupation and geographic locations (*P* < 0.05). These differences were mainly reflected in the prevalence of CRF01_AE, CRF07_BC, CRF08_BC and subtype B. A relatively higher prevalence of CRF08_BC (23.0%, 17/74) was found in females. Figure 3 depicts the trends of HIV-1 genotypes across HIV confirmation year, it indicated that the prevalence of subtype B and CRF08_BC decreased, while the prevalence of CRF01_AE gradually increased over time (trend *χ*^*2*^ = 6.768, *P* < 0.05). By comparing the corresponding results before 2008 and after 2012, it indicated that subtype B decreased from 33.3% (8/24) to 7.0% (7/100), CRF08_BC decreased from 16.7% (4/24) to 9% (9/100), while CRF01_AE increased from 16.7% (4/24) to 52% (52/100). Among farmers, a relatively lower prevalence of CRF01_AE (25.8%, 8/31), but a higher prevalence of subtype B (29.0%, 9/31) and CRF08_BC (25.8%, 8/31) were found detected. As shown in Fig. 1, HIV-1 genotype varied among different geographic locations of Jiangsu. A relatively higher prevalence of CRF07_BC (31.4%, 27/86) and subtype B (19.5%, 16/82) were found in Central Jiangsu and South Jiangsu, respectively. A relatively lower prevalence of subtype B (7.0%, 6/86) and CRF08_BC (7.0%, 6/86) was observed in Central Jiangsu.

## Discussion

In this study, HIV-1 genotypes circulating among the heterosexually transmitted population in Jiangsu was investigated based on analyses of HIV-1 *gag* and *env* gene sequences. The results showed that CRF01_AE (45.4%), CRF07_BC (21.4%), subtype B (12.6%) and CRF08_BC (11.1%) were dominant among the heterosexual transmitted HIV-1 population in Jiangsu, which is in accordance with data among heterosexual HIV transmissions in the neighboring provinces (Shanghai, Zhejiang, and Anhui) [16–18]. However, in contrast to the previous finding in Jiangsu [8], this study finds that the prevalence of different HIV-1 subtypes has changed continually from 2006 to 2017, especially for CRF01_AE (from 20.9 to 45.4%), subtype B (from 23.3 to 12.6%) and CRF07_BC (from 32.6 to 21.4%). Changes in the relative prevalence of genotype illustrate a shift in HIV transmission patterns. In Jiangsu between 1991 and 1998, HIV-1 infection was initially confined to IDUs, FPDs and recipients of blood transfusions. However, HIV-1 infection cases increased rapidly in the following decades, such that sexual transmission has overtaken other transmission routes as the predominant transmission pathway of the HIV-1 epidemic. Consequently, instead of the previously pure subtype B, CRF01_AE has become the main strain of HIV-1 infection among heterosexuals. This change in transmission routes is also reflected in the observed change in prevalence of the subtypes across HIV confirmation times. Additionally, the result showed a relatively higher prevalence of CRF07_BC (31.4%) in Central Jiangsu and subtype B (19.5%) in South Jiangsu. This may be attributed to the region’s rapid economic development. Indeed, a previous report indicated that nearly 70% of HIV-positive cases were living in South Jiangsu [19], all in cities located in high speed railway or highway lines crossing Jiangsu with relatively higher economic growth than other areas, these may provide a direct or indirect facilitation for fast population mobility and HIV spreading.

HIV-1 subtypes distribution had previously been synchronized with risk-specific groups in China. For example, subtype B is the main subtypes among FDPs in central China [20], CRF07_BC and CRF08_BC is dominant among IDUs in southwestern China [21, 22], while CRF01_AE, CRF07_BC and subtype B are the major strains circulating in MSM in eastern China including Jiangsu province [5, 23, 24]. The HIV subtypes epidemic in heterosexuals in this study are somewhat similar to those reported in MSM in previously published literature. Although it is not certain that these subtypes are transmitted from MSM to heterosexual individuals, the findings of this study support this theoretical vector route and warrant our close attention. Additionally, unlike the restrained distribution of HIV-1 subtypes in other specific high-risk groups, broad HIV-1 genetic diversity was found among heterosexuals. This study identified multiple subtypes, including CRF01_AE, CRF07_BC, CRF08_BC, subtype B, subtype C, previous rarely reported CRF68_01B, CRF55_01B, CRF02_AG, subtype A and some URFs, implying that the HIV genotypes prevalent in HIV-infected individuals via heterosexual transmission are complex and diverse in this area.

This study revealed that CRF01_AE was the most prevalent HIV-1 strains among heterosexuals (43.86%) in Jiangsu. In recent years, the persistence of multiple lineages of HIV-1 CRF01_AE strains indicated that certain transmission networks might facilitate the transmission of HIV-1 [25, 26]. Of note, previous studies indicated that patients infected with CRF01_AE had a faster progression to AIDS than with other HIV-1 subtypes, which is associated with a high proportion of X4 tropism [27, 28]. Cumulative evidences on newly diagnosed individuals infected by HIV-1 CRF01_AE strain suggest that CRF01_AE is most prevalent in early infections [29–31]. As the predominant subtype among heterosexuals, it can be hypothesized that CRF01_AE might remain the primary subtype and increase the HIV disease burden in Jiangsu in the coming years. Therefore, there is a pressing need to take further steps to limit the epidemic of this CRF.

In recent years, many novel HIV-1 recombinant strains in heterosexuals have since been confirmed by using molecular subtyping in China [32–34], suggesting that heterosexual transmission is intermixed with all other high-risk populations and contribute to the complexity of the HIV-1 epidemic. In this study, several potential HIV-1 recombinant strains may be present in some subjects. Most participants reported more than 2 heterosexual partners, creating opportunity for co-circulation and dual infection with different subtypes/CRFs of HIV-1, allowing them to mingle and recombine. Thus, research into novel HIV-1 recombinant forms should be a priority for future studies. Additionally, individuals in our study who reported contact with commercial sex workers were particularly vulnerable for HIV infection. They also play a critical role in bridging the epidemic to general population via sex contact with clients and regular sexual partners [35]. The questionnaire survey in this study showed that about 45% of enrolled HIV infections in this study were related with commercial heterosexual services. Due to low incomes and work-life pressures, some people seek low-cost commercial sex services, which increases the risk of HIV infection. We believe that this results should increase alarm regarding the spread of HIV, and indicates a greater need to control this epidemic.

This study has several limitations. First, risk assessment and attribution may be subject to biases, as some subjects might be unwilling to disclose their risk status. In this regard, a recently developed HIV-1 transmission network analysis based on highly similar HIV-1 genetic sequences might provide empirical evidence to identify the transmission source [36–38]. Second, only samples with viral load > 1000 copies/mL were included in this study to account for amplification efficiency. The scope of future research should be expanded to comprehensively outline HIV genetic profiles in this area including viral load < 1000 copies/mL. Finally, samples with different genotypic results from the *gag* and *env* gene regions were provisionally defined as URFs. Further analyses of the near full-length genome of HIV-1 is required.

## Conclusion

In conclusion, our study demonstrated that CRF01_AE was the most predominant HIV-1 subtypes among the heterosexually transmitted population in Jiangsu, followed by CRF07_BC, subtype B, and CRF08_BC, as well as the previously minor CRF68_01B and subtype C. Extensive HIV-1 genetic diversity suggests that HIV epidemic in this population is complex and severe. Consequently, there is an urgent need to implement more effective and integrated intervention programs to limit the further dissemination of HIV-1.
